# Stress of Overseas Long-Distance Care During COVID-19: Potential “CALM”ing Strategies

**DOI:** 10.3389/fpsyt.2021.734967

**Published:** 2021-10-01

**Authors:** Aparna Das, Kalpana P. Padala, Prabhava Bagla, Prasad R. Padala

**Affiliations:** ^1^Department of Psychiatry, University of Arkansas for Medical Sciences, Little Rock, AR, United States; ^2^Geriatric Research Education, and Clinical Center, Central Arkansas Veterans Healthcare System, Little Rock, AR, United States; ^3^Department of Geriatrics, University of Arkansas for Medical Sciences, Little Rock, AR, United States; ^4^Department of Internal Medicine, AdventHealth Orlando, Orlando, FL, United States

**Keywords:** COVID-19, family members, immigrants, physical distance, health

## Abstract

“CALM”ing strategies during COVID-19 pandemic. Created with BioRender.com. 
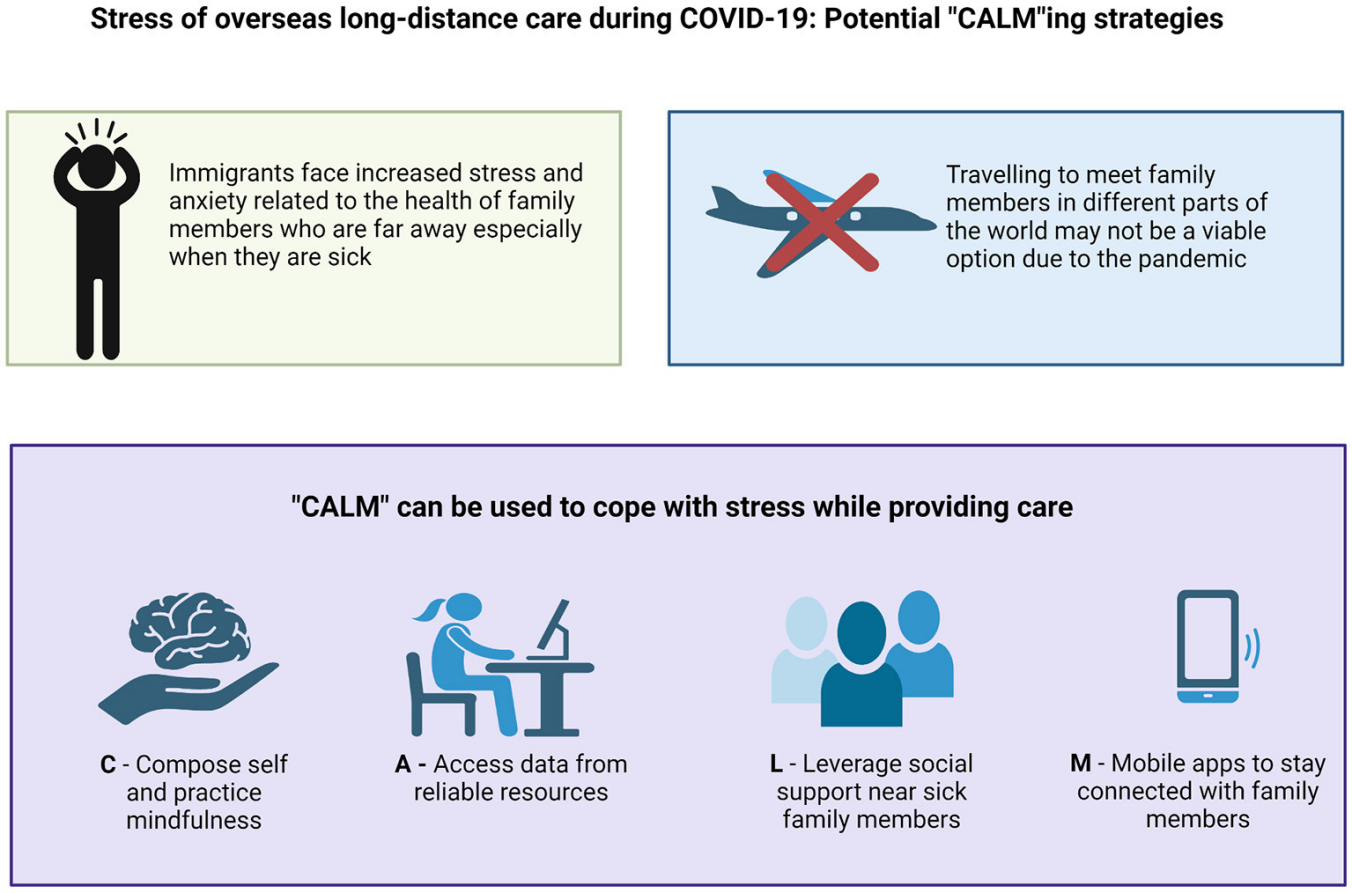

Coronaphobia, a fear of contracting COVID-19, has been a particular problem for immigrants in the US whose families live in their native lands (1). The United States is a land of immigrants with immigrants and their US–born children number constituting ~26 percent (around 85.7 million) of the population (2). There has been an increase in stress and anxiety in association with the ongoing pandemic, and immigrants are not immune to it. Although measures have been taken to control the spread, and new vaccines are being developed and administered, fear continues to the grip the entire world. With new strains creeping in, it only adds to more anxiety and uncertainties to the already existing undercurrent of tension related to COVID-19.

Immigrants are concerned for the safety of the have family members in their home country and the well-meaning worries related to health of family members have escalated during the pandemic. Their parents and most of their close family members back home are in the older adult age group which makes them more vulnerable to COVID-19 infection. Additionally, if they have medical comorbidities, then they are stratified to be in the high-risk category for COVID-19 infection. In the current setting of the pandemic, when any family member back home reports that they are sick (especially with flu-like symptoms), it creates anxiety, stress, and unrest in the immigrant who is thousands of miles away in the US. The first impulse is to think about COVID-19 infection as that is the viral infection that is the most prevalent in current times. Other factors that add to existing stress and anxiety are inability to get tested in other countries due to limited resources, or test avoidance by family members due to various reasons like misinformation, stigma or financial barriers.

The immigrants' parents and other family members cannot be in the US for various reasons, such as personal preference, visa, and travel restrictions. For immigrants, traveling to their home country is an arduous process and may not be an option currently for a multitude of reasons. It requires preparation in advance, extended time off and may need half to a full day to travel across the globe. It is expensive and may lead to loss of productivity and utilization of unpaid leaves which leads to a financial burden. The pandemic has made travel even more challenging as there is the risk of getting infected with this deadly virus. Unlike pre-COVID era when people could travel to their home countries and visit family members, travel has not remained a viable option. Even if one considers risking travel, one has to jump through hoops to reach the final destination such as adhering to different travel guidelines and policies which are variable and frequently changing, taking a longer route as there is travel ban in some countries to limit the entry of the new strain of virus. If that is tackled and somehow one is successful in reaching home, then the first few days upon arrival may be spent in quarantine (3). If COVID negative test results are not provided, little can be done to help sick family members in that period. The question arises as how to tackle this situation, which is nerve-wracking in more ways than one.

The surge of variants, in particular delta variant has created fresh challenges. It has shown that even fully vaccinated people are susceptible to the infection. Such breakthrough infections appear rare and mild (4) but scientific data remains limited. Even in places with relatively high vaccination coverage (5), fully vaccinated people run the risk of getting infected in large gatherings, leading to guidance that social distancing and mask-wearing continue regardless of vaccination status. Although a direct comparison is not appropriate due to different vaccines being available in different countries, at least one study (6) noted fully vaccinated individuals to be infected in similar numbers as those who are unvaccinated albeit experience a minor course of illness. Such news taken out of context, might affect the morale of the general population and their confidence in vaccines and other preventative measures.

Considering the common hurdles, emotional reaction and available remote resources we came up with an approach to allay anxiety for the immigrants while helping their loved ones actively. Immigrants can consider a few things to navigate through this situation. We have devised the following mnemonic to help immigrants who are physically separated from family members deal with this situation: CALM.

C–Compose self and act mindfully. It is common to feel terrified when a relative who is far away has a fever or is otherwise sick, due to the variability of presentation and high prevalence of COVID-19. However, it is important to realize that there could be other reasons for illness, such as common cold. Many illnesses can be promptly cured, and, not all illnesses are deadly. This knowledge may allay some concern about COVID-19 but there may be lingering anxiety which could be reduced by mindfulness. Several studies have reported that mindfulness-based interventions are beneficial in reducing anxiety, depression, and stress levels (7–9). Apart from helping with stress, and anxiety in healthy population, mindfulness has been shown to be helpful for patients with chronic illnesses to cope well (10).

There are many mindfulness apps available like The Mindfulness App, Headspace, Calm, Serenity etc, which can come in handy. The authors recommend trying different platforms before deciding the one that works for an individual. Once they decide and pick the one, they feel is the best for them, sticking with it and practicing regularly for around 10–30 min daily will lead to optimal benefits. Many of these apps are available for iOS and Android platforms and have free tiers that can be utilized to assess their suitability. They offer a variety of meditation sessions of varying durations. The benefits of such apps might also extend to patients as well, and immigrants might find it beneficial to coordinate a time with their loved ones overseas to simultaneously try a meditation session and discuss outcomes.

A–Access data from reliable resources. The world wide web is full of data and information about COVID-19, but all information may not be accurate. Reliance on dubious news articles or shows which might be sensationalizing the situation, or on word-of-mouth personal experiences may often magnify the stress. Reviewing websites like Centers for Disease Control and Prevention (CDC) (11) and The Institute for Health Metrics and Evaluation (IHME) (12) can provide a much more reliable data. IHME has country-specific data which immigrants can use to better understand the situation in their home country. It may be that the situation in a particular place is not as bad as the immigrant automatically assumes.

L–Leverage social network around the sick family member. Social network can be very supportive in these testing times. There is evidence that higher level of perceived social support helps in counteracting the negative impact of the pandemic on mental health and helps in building resilience which can help in quicker recovery (13). Social network can be considered within micro-context (e.g., family members), and macro-context (e.g., community-based networks) (13). In many cultures, extended families live relatively close to each other. Using their help in such a situation can prove vital. They are physically present near the index family member and can provide physical and emotional support. In some cases, friends and co-workers may also support the sick family member by helping them with food, medicines, and if needed arranging a doctor's visit, while taking the necessary precautions to protect themselves from COVID or other communicable infections. They may also be able to give a better and unbiased account of the health of the involved family members to the immigrant. Also, people in the vicinity are more aware of the local resources available e.g., hospitals providing COVID specific care, to help deal with this situation.

M–Mobile apps like Google Hangouts, Duo, Facetime, WhatsApp, and Signal, can be used to communicate with family members abroad. The texting, audio, and video services available with these apps have made communication easier and are great ways of providing family-centered care during the pandemic (14, 15). Communicating with family members who are at distant places via audio or video may give an appraisal of their general health condition, which may help allay some anxiety. These apps are encrypted and can be used to share protected health information such as results of labs, imaging etc. with the consent of sick family members. This information can be helpful to some immigrants, especially those who are health care professionals. Reviewing such information can give a fair idea of the condition and the type of medical care being provided, which can be reassuring. If care provided is not optimal, then redirecting them to better resources may be done. Frequent communication with the sick family member also gives the family member a feeling of comfort and being cared for. This may help in strengthening the overall bond among family members.

In conclusion, the COVID-19 pandemic has brought with it many challenges and illness of close family members who are separated by distance only adds more stress and feelings of helplessness to the already overwhelming situation. Travel is risky and may not be advisable. Using CALM techniques may help immigrants and families allay some anxiety and help them deal with this trying situation.

## Author Contributions

AD: conceptualization, literature review, interpreting data, and manuscript preparation. KP: critical review of the manuscript and manuscript preparation. PB: literature review and critical review of the manuscript. PP: conceptualization, critical review, and manuscript preparation. All authors have contributed significantly to the paper and approval of the final version.

## Conflict of Interest

The authors declare that the research was conducted in the absence of any commercial or financial relationships that could be construed as a potential conflict of interest.

## Publisher's Note

All claims expressed in this article are solely those of the authors and do not necessarily represent those of their affiliated organizations, or those of the publisher, the editors and the reviewers. Any product that may be evaluated in this article, or claim that may be made by its manufacturer, is not guaranteed or endorsed by the publisher.

## References

[1] Dubey S Biswas P Ghosh R Chatterjee S Dubey MJ Chatterjee S. Psychosocial impact of COVID-19. Diabetes Metab Syndr. (2020) 14:779–88. 10.1016/j.dsx.2020.05.035 32526627 PMC7255207

[2] Migration Policy Institute (MPI). Available online at: https://www.migrationpolicy.org/article/frequently-requested-statistics-immigrants-and-immigration-united-states-2020 (accessed June 28, 2021).

[3] Bielecki M Patel D Hinkelbein J Komorowski M Kester J Ebrahim S. Reprint of: Air travel and COVID-19 prevention in the pandemic and peri-pandemic period: a narrative review. Travel Med Infect Dis. (2020) 38:101939. 10.1016/j.tmaid.2020.101939 33291000 PMC7831384

[4] Bergwerk M Gonen T Lustig Y Amit S Lipsitch M Cohen C. Covid-19 breakthrough infections in vaccinated health care workers. N Engl J Med. (2021). [Epub ahead of print]. 10.1056/NEJMoa2109072 34320281 PMC8362591

[5] Brown CM Vostok J Johnson H Burns M Gharpure R Sami S. Outbreak of SARS-CoV-2 infections, including COVID-19 vaccine breakthrough infections, associated with large public gatherings–Barnstable County, Massachusetts, July 2021. MMWR Morb Mortal Wkly Rep. (2021) 70:1059–62. 10.15585/mmwr.mm7031e2 34351882 PMC8367314

[6] Thangaraj JWV Yadav P Kumar CG Shete A Nyayanit DA Rani DS. Predominance of delta variant among the COVID-19 vaccinated and unvaccinated individuals, India, May 2021. J Infect. (2021) 6:S0163-4453(21)00387-X. 10.1016/j.jinf.2021.08.006 34364949 PMC8343391

[7] Chiesa A Serretti A. Mindfulness-based stress reduction for stress management in healthy people: a review and meta-analysis. J Altern Complement Med. (2009) 15:593–600. 10.1089/acm.2008.0495 19432513

[8] Hofmann SG Sawyer AT Witt AA Oh D. The effect of mindfulness-based therapy on anxiety and depression: a meta-analytic review. J Consult Clin Psychol. (2010) 78:169–83. 10.1037/a0018555 20350028 PMC2848393

[9] Goldberg SB Tucker RP Greene PA Davidson RJ Wampold BE Kearney DJ. Mindfulness-based interventions for psychiatric disorders: a systematic review and meta-analysis. Clin Psychol Rev. (2018) 59:52–60. 10.1016/j.cpr.2017.10.011 29126747 PMC5741505

[10] Howarth A Perkins-Porras L Copland C Ussher M. Views on a brief mindfulness intervention among patients with long-term illness. BMC Psychol. (2016) 4:56. 10.1186/s40359-016-0163-y 27842610 PMC5109661

[11] Centers for Disease Control and Prevention (CDC). Available online at: https://www.cdc.gov/coronavirus/2019-ncov/symptoms-testing/symptoms.html (accessed June 28, 2021).

[12] Institute for Health Metrics and Evaluation (IHME). Available online at: https://covid19.healthdata.org/global?view=total-deaths&tab=trend (accessed June 30, 2021).

[13] Li F Luo S Mu W Li Y Ye L Zheng X. Effects of sources of social support and resilience on the mental health of different age groups during the COVID-19 pandemic. BMC Psychiatry. (2021) 21:16. 10.1186/s12888-020-03012-1 33413238 PMC7789076

[14] Papadimos TJ Marcolini EG Hadian M Hardart GE Ward N Levy MM. Ethics of outbreaks position statement. Part 2: Family-centered care. Crit Care Med. (2018) 46:1856–60. 10.1097/CCM.0000000000003363 30312225

[15] Voo TC Senguttuvan M Tam CC. Family presence for patients and separated relatives during COVID-19: physical, virtual, and surrogate. J Bioeth Inq. (2020) 17:767–72. 10.1007/s11673-020-10009-8 32840835 PMC7445690

